# The Role of the Delta Neutrophil Index, Pan‐Immune Inflammation Value and Other Hematologic Inflammatory Parameters in the Preliminary Diagnosis of Tubal Ectopic Pregnancy

**DOI:** 10.1002/iid3.70349

**Published:** 2026-02-05

**Authors:** Şeyma Nur Karadag, Dilay Gök Korucu

**Affiliations:** ^1^ Department of Obstetrics and Gynecology University of Health Sciences Konya City Hospital Konya Turkey; ^2^ Department of Obstetrics and Gynecolog, IVF Unit University of Health Sciences Konya City Hospital Konya Turkey

**Keywords:** delta neutrophil index, early diagnosis, ectopic pregnancy, hematologic markers, inflammation

## Abstract

**Background:**

Differentiating between healthy intrauterine pregnancy (HP) and ectopic pregnancy (EP) in early gestation is clinically challenging.

**Aim:**

This study aimed to evaluate the role of the delta neutrophil index (DNI), pan‐immun inflammation value (PIV) and other hematologic inflammatory markers in the preliminary diagnosis of tubal EP. To the best of our knowledge, there are no studies in the literature examining the relationship between DNI, PIV and EP.

**Materials and Methods:**

This retrospective study included 120 women diagnosed with tubal EP and 102 women with HP who presented to a tertiary hospital in Turkey between 2019 and 2023. Inclusion criteria were women with confirmed tubal EP or HP who had complete clinical and hematologic data and no chronic diseases, infections, or body mass index (BMI) > 30 kg/m^2^. The primary outcome was to assess whether DNI, PIV and other complete blood count (CBC) parameters could differentiate EP from HP. Statistical analysis included *t*‐tests, Welch's *t*‐test, *χ* ^2^ tests, and ROC analysis; *p* < 0.05 was considered statistically significant.

**Results:**

No significant difference was found in DNI value (*p* = 0.256). However, white blood cell count (WBC) (*p* = 0.007), platelet (PLT) (*p* = 0.001), lymphocyte (*p* < 0.001), PIV (*p* = 0.023), systemic immune‐inflammation index (SII) (*p* = 0.016) and basophil (*p* = 0.005) levels were higher in the EP group, while hemoglobin (HB) levels were lower (*p* < 0.001).

**Conclusion:**

While DNI was not a significant marker, other hematologic parameters may support early identification of EP, suggesting an underlying inflammatory process.

## 1 Introduction

Ectopic pregnancy (EP) is defined as the implantation of a fertilized ovum outside the uterine cavity, most commonly in the fallopian tubes. It is a significant cause of maternal morbidity and mortality during the first trimester [1]. Known risk factors include a history of EP, assisted reproductive techniques, pelvic infections, tubal surgery, and intrauterine device use [2, 3].

Despite advancements in ultrasonography and serum beta‐human chorionic gonadotropin (β‐hCG) testing, early diagnosis of EP remains challenging in some cases. A delayed or missed diagnosis can lead to tubal rupture, hemorrhage, and maternal death. In particular, differentiating EP from normal intrauterine pregnancy in the absence of definitive imaging findings is a diagnostic dilemma. Therefore, clinicians have sought additional laboratory parameters that may help in the early identification of EP without the need for invasive procedures.

Recent evidence suggests that inflammation plays a role in the pathogenesis of EP. Inflammatory markers derived from routine CBC such as the WBC, neutrophil‐to‐lymphocyte ratio (NLR), platelet‐to‐lymphocyte ratio (PLR), and DNI have been studied in various obstetric conditions [4, 5]. The DNI reflects the proportion of immature granulocytes in peripheral blood and has been investigated as a marker of systemic inflammation in sepsis, preeclampsia, and appendicitis [6, 7]. However, no prior study has evaluated its diagnostic role in EP.

Therefore, this study aimed to assess whether DNI, PIV and other hematologic parameters may be useful in the preliminary diagnosis**—**that is, the early clinical suspicion of EP prior to definitive radiologic or surgical confirmation. Identifying cost‐effective and easily accessible markers could improve early management and outcomes.

## 2 Materials and Methods

This retrospective study was conducted after obtaining approval from Konya Karatay University's Medical Faculty Non‐Interventional Clinical Research Ethics Committee (Decision No: 2024/045, dated June 6, 2024). All procedures were conducted in accordance with the Declaration of Helsinki, as revised in 2000. Informed written and verbal consent was obtained from all participants. A total of 222 patients were included: 120 women diagnosed with tubal ectopic pregnancy (EP group) and 102 women with healthy intrauterine pregnancies resulting in live births (HP group). All participants presented to the Obstetrics and Gynecology Department of University of Health Sciences Konya City Hospital between 2019 and 2023.

The cases (EP group) and controls (HP group) were selected retrospectively from the hospital database. No formal matching was performed between the groups. However, the same inclusion and exclusion criteria were applied to both groups to minimize potential confounding. Inclusion criteria consisted of women with a confirmed diagnosis of tubal ectopic pregnancy or healthy intrauterine pregnancy in the first trimester, with complete clinical and laboratory data. Patients were excluded if they had chronic diseases (e.g., hypertension, thyroid disorders, diabetes mellitus, inflammatory or autoimmune diseases), signs of infection, a history of smoking or alcohol use, or a BMI greater than 30 kg/m².

The diagnosis of EP was based on measurement of serial β‐hCG levels and transvaginal ultrasound findings, with histopathologic confirmation. For the HP group, blood samples were collected during routine antenatal visits between the 6th and 8th weeks of gestation. For the EP group, blood samples were obtained upon admission and diagnosis.

Demographic and obstetric data were extracted from patient records, and laboratory values were retrieved from the electronic medical system. The following parameters were evaluated: age, gravida, parity, BMI, HB, WBC, PLT count, neutrophils, lymphocytes, monocytes, eosinophils, basophils, and DNI. Additional inflammatory indices were calculated, including neutrophil‐to‐lymphocyte ratio (NLR), platelet‐to‐lymphocyte ratio (PLR), monocyte‐to‐lymphocyte ratio (MLR), systemic inflammation response index (SIRI), SII and PIV.

### 2.1 CBC Laboratory Calculations

CBC analyzes were performed on Sysmex XN‐1000 Automated Hematology System (Hematology System, Japan). WBC, neutrophil count, lymphocyte count, monocyte count, basophil, PLT count, values were obtained from CBC analyzes. In our hospital, the serum DNI level is regularly determined as part of a CBC via an automated cell analyzer. It is derived by subtracting the fraction of mature polymorphonuclear neutrophils measured via a myeloperoxidase channel from the total neutrophil count obtained through a nuclear lobularity channel.

The following formulas were used for the values obtained through calculations, respectively [8, 9]:

Neutrophiltolymphocyteratio(NLR)=neutrophilcount/lymphocytecount;

Platelettolymphocyteratio(PLR)=plateletcount/lymphocytecount;

Monocytetolymphocyteratio(MLR)=monocytecount/lymphocytecount

Systemicimmune‐inflammationindex(SII)=plateletcountxneutrophilcount/lymphocytecount.

Systemicinflammationresponseindex(SIRI)=(Neutrophils×Monocytes)/Lymphocytes

dNLR:Neutrophil/(WBC‐Neutrophil)

NLPR=Neutrophil/LymphocytexPlatelet

Pan‐immune‐inflammationvalue(PIV)=(Neutrophils×Monocytes×Platelets)/Lymphocytes

### 2.2 Statistical Analysis

Statistical analysis was performed using IBM SPSS version 27. Data distribution has been evaluated for skewness and kurtosis, and skewness and kurtosis coefficients within the range of ± 2 are considered evidence of a normal distribution [10]. However, with a sample size of this magnitude, parametric tests are robust to moderate deviations from normality. Variables are presented as mean ± SD. Comparisons between groups were conducted using independent samples *t*‐test or Welch's *t*‐test, as appropriate. *χ* ^2^ or Fisher's exact test was used for categorical data. ROC analysis was used to identify potential diagnostic cut‐off values. A *p*‐value < 0.05 was considered statistically significant. Effect sizes were calculated using Cramer's V for Pearson's *χ* ^2^ test and Cohen's *d* value for independent groups *t*‐test and Welch's *t*‐test. Cohen's *d* value indicates small effect sizes of *d* = 0.20, medium effect size of *d* = 0.50, and large effect size of *d* = 0.80 [11].

## 3 Results

A total of 222 patients were included in the study: 120 with confirmed tubal ectopic pregnancy (EP group) and 102 with healthy intrauterine pregnancy resulting in live birth (HP group). Table 1 presents the comparison of demographic and clinical characteristics between the two groups.

**Table 1:** Comparison of EP history and numerical data of participants by pregnancy status (*N* = 222).

		Group 2 (HP) (*N* = 102)	Group 1 (EP) (*N* = 120)	Statistical analysis		Effect Size
		*N* (%)^b^	*N* (%)^b^	*χ* ^2^	*p* *	Cramer's V
History of EP	No	102 (100.0)	107 (89.2)	12.40	0.001	0.23
	1 Ectopic Pregnancy	0 (0.0)	10 (8.3)			
	2 Ectopic Pregnancies	0 (0.0)	3 (2.5)			
Numerical data				***t***	***p*** **	**Cohen's *d***
Age (years)	x±SD	27.11 ± 5.15	30.16 ± 4.90	−4.52	< 0.001	0.61
Number of pregnancies (*n*)	x±SD	2.20 ± 1.14	3.17 ± 1.63	−5.20^a^	< 0.001	0.68
Number of deliveries (*n*)	x±SD	0.96 ± 0.92	1.46 ± 1.17	−3.55^a^	< 0.001	0.47
		***N*** = **86**	***N*** = **91**	***t***	***p*** **	**Cohen's d**
BMI(kg/m^2^)	x ± SD	24.66 ± 3.29	24.50 ± 2.72	0.33	0.740	0.05

* Fisher Freeman Halton test,

** Independent groups *t*‐test,

^a^ Welch's *t*‐test,

^b^ Column percentage HP: Healthy pregnancy, EP: Ectopic pregnancy, BMI: Body mass index.

Patients in the EP group were significantly older than those in the HP group (30.16  ±  4.90 vs. 27.11  ±  5.15 years; *p* <  0.001). Gravidity and parity were also significantly higher in the EP group (*p* <  0.001 for both). No significant difference was observed in BMI between the groups (*p* =  0.740). A history of previous EP was present in 10.8% of the EP group and in none of the HP group (*p* =  0.001).

Laboratory findings are summarized in Table 2. The EP group had significantly higher levels of WBC (9.11  ±  2.87 vs. 7.96  ±  1.37 × 10³/μL, *p* <  0.001), PLT (279.63  ±  65.72 vs. 253.15  ±  54.51 × 10³/μL, *p* =  0.001), lymphocytes (2.25 ±  0.77 vs. 1.96 ±  0.43 ×10³/μL, *p* <  0.001), basophils (0.03  ±  0.02 vs. 0.02  ±  0.02 ×10³/μL, *p*  =  0.005), PIV (393 ± 205 EP vs. HP 490 ± 408; *p* = 0.023) and SII (*p* = 0.016). HB levels were significantly lower in the EP group (12.39  ± 1.22 vs. 13.21  ±  0.73 g/dL, *p* <  0.001). There was no significant difference between the groups in DNI value (0.32 ± 0.14% in EP vs. 0.34 ± 0.17% in HP; *p* =  0.256).

**Table 2:** Comparison of laboratory values by pregnancy status (*N* = 222).

Laboratory results		HP (*N* = 102)	EP (*N* = 120)	*t*	*p* *	Cohen's *d*
HB g/dl	x ± SD	13.21 ± 0.73	12.39 ± 1.22	6.15	< 0.001	0.80
PLT 10^3^/µL	x ± SD	253.15 ± 54.51 10^3^	279.63 ± 65.72	−3.23	0.001	0.44
Lymphocyte 10^3^/µL	x ± SD	1.96 ± 0.43 10^3^	2.25 ± 0.77	−3.57	< 0.001	0.46
Basophil 10^3^/µL	x ± SD	0.02 ± 0.02 10^3^	0.03 ± 0.02	−2.86	0.005	0.39
WBC 10^3^/µL	x ± SD	7.96 ± 1.37 10^3^	9.11 ± 2.87	−3.88^a^	< 0.001	0.50
Neutrophil 10^3^/µL	x ± SD	5.35 ± 1.11 10^3^	6.16 ± 2.91	−2.83^a^	0.005	0.36
Eosinophil 10^3^/µL	x ± SD	0.11 ± 0.10 10^3^	0.12 ± 0.13	−0.97^a^	0.332	0.13
Monocyte 10^3^/µL	x ± SD	0.53 ± 0.14 10^3^	0.56 ± 0.18	−1.22^a^	0.223	0.16
NLR	x ± SD	2.85 ± 0.80	3.36 ± 3.10	−1.76^a^	0.080	0.22
PLR	x ± SD	134 ± 35.8	142 ± 68.5	−1.03^a^	0.303	0.13
MLR	x ± SD	0.29 ± 0.12	0.27 ± 0.09	1.50^a^	0.134	0.21
SIRI	x ± SD	1.54 ± 0.69	1.71 ± 1.18	−1.36^a^	0.176	0.18
SII	x ± SD	722 ± 273	964 ± 1049	−2.43^a^	0.016	0.31
DNI	x ± SD	0.34 ± 0.17%	0.32 ± 0.14	1.14^a^	0.256	0.16
DNLR	x ± SD	2.10 ± 0.51	2.50 ± 2.26	−1.91^a^	0.059	0.24
NLPR	x ± SD	1.24 ± 0.51	1.27 ± 1.19	−0.26^a^	0.793	0.03
PIV	x ± SD	393 ± 205	490 ± 408	−2.29^a^	0.023	0.29

Abbreviations: DNLR, derived neutrophil‐lymphocyte ratio; EMR, eosinophil/monocyte ratio; MLR, monocyte/lymphocyte ratio; NLPR, neutrophil (lymphocyte × platelet) ratio; NLR, neutrophil/lymphocyte ratio; PLR, platelet/lymphocyte ratio; PIV, pan‐immune inflammation value; SII, systemic immune inflammation index; SIRI, systemic inflammatory response index.

* Independent groups *t*‐test.

^a^ Welch's *t*‐test.

When the effect sizes were examined, Cramer's *V* value was 0.23, indicating a medium effect size. According to Cohen's *d* values, the number of pregnancies showed a medium‐to‐large effect across groups (*d* = 0.68), age (*d* = 0.61) and the number of deliveries (*d* = 0.47) had medium effects, while BMI exhibited a very small effect (*d* = 0.05). HB demonstrated a large effect on group differences (*d* = 0.80), whereas PLT count (*d* = 0.44), lymphocyte count (*d* = 0.46), basophil count (*d* = 0.39), and WBC count (*d* = 0.50) showed medium effects. Neutrophil count (*d* = 0.36), SII (*d* = 0.31), and PIV (*d* = 0.29) exhibited small‐to‐medium effects, while the remaining parameters showed small effects (Tables 1 and 2).

**Figure 1:**
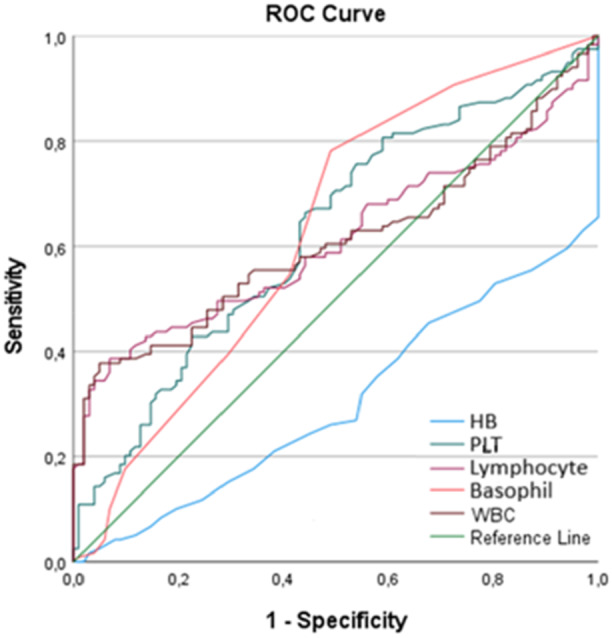
ROC curve.

To assess diagnostic potential, ROC curve analysis was performed for variables with significant differences between the groups (HB, WBC, PLT, lymphocyte, and basophil). AUC values were as follows: WBC (0.605), PLT (0.625), lymphocyte (0.608), basophil (0.630), and HB (0.301), as shown in Table 3 and Figure 1. Although the AUC values were relatively low and did not indicate strong diagnostic accuracy, they may still provide supportive information as part of the initial clinical assessment of suspected EP.

**Table 3:** ROC analysis * Results of the ROC analysis conducted to determine the cutoff values of HB, PLT, lymphocyte, basophil, and WBC for participants by group.

	AUC	Cut off	Sensitivity	Specificity	*p* value
WBC (µL)	0.605	7.57 10^3^/µL	65%	35.3%	0.007
HB (µL)	0.301	12.45 g/dl	52.5%	19.6%	0.001
PLT (µL)	0.625	248.500 10^3^/µL	70%	51%	0.001
Basophil (µL)	0.630	0.015 10^3^/µL	78.2%	51%	0.001
Lymphocyte (µL)	0.608	1.82 10^3^/µL	67.5%	43.1%	0.006

## 4 Discussion

EP accounts for approximately 1%–2% of all pregnancies and is a leading factor causing pregnancy‐related maternal mortality in the first trimester despite advancements in diagnostic and treatment methods [10, 12]. EP is responsible for 2.7% of all maternal deaths [13]. Thanks to recent advancements, high‐resolution transvaginal ultrasonography and β‐hCG tests allowed for early and accurate diagnosis. However, there still are cases where diagnosis remains challenging, and the exact location of the EP is unknown [12, 14].

In this study, HB, PLT, lymphocyte, basophil count, SII, PIV, and WBC values significantly differed by pregnancy status. The mean HB level in the HP group was higher (*p* < 0.001), while EP group had higher mean WBC (*p* = 0.007), PLT (*p* = 0.001), lymphocyte (*p* < 0.001), basophil (*p* = 0.005), SII (*p* = 0.016) and PIV (*p* = 0.023) levels. However, there were no significant differences regarding DNI value (*p* = 0.256).

A meta‐analysis of 12 studies reported that DNI holds significant diagnostic value in infection and mortality settings [15]. Other studies have also highlighted its prognostic role in surgical outcomes and disease severity, including tubo‐ovarian abscess and acute cholecystitis [16, 17]. However, our study did not demonstrate a significant diagnostic utility of DNI in the preliminary assessment of EP. This may be partly due to the notable age difference between groups, which could have influenced inflammatory parameters. Further studies with larger and demographically balanced populations are warranted to evaluate its potential role in EP.

Recent studies have demonstrated the prognostic utility of PIV in various clinical settings. Uçar et al. [18] reported that higher pretreatment PIV scores were associated with poorer prognosis in patients with locally advanced rectal cancer. Similarly, Uçaner et al. [19] showed that PIV could aid in predicting the need for surgery in adhesive small bowel obstruction, highlighting its value in clinical decision‐making during surgical emergencies. However, no previous studies have evaluated the diagnostic or prognostic role of PIV in EP. In our study, PIV values were assessed higher in the EP group (*p* = 0.023). This finding further substantiate the concept that EP is accompanied by a pronounced inflammatory response, highlighting the pivotal role of inflammation in its underlying pathophysiology. Further studies with larger and demographically balanced populations are warranted to clarify the potential diagnostic relevance of PIV in EP.

Regarding the mean age, the mean age of patients with EP was determined to be higher. Age is a known risk factor for EP, and the results achieved in this study align with the literature, confirming that patients diagnosed with EP tend to be older than those with normal pregnancies [20]. Additionally, the EP history was reported to be significantly higher in the EP group. Given that EP history is a well‐established risk factor, this finding is consistent with the literature [21].

When comparing WBC levels, EP group was found to have higher WBC levels (*p* < 0.001), in line with the literature. Previous studies reported similar findings. Abdullah et al. reported significant differences in WBC levels not only between the EP group and HP women but also between ruptured and non‐ruptured ectopic pregnancies [4]. The WBC levels were higher in the EP group and were even higher in the ruptured EP subgroup than in the non‐ruptured subgroup. This suggests that elevated WBC levels may be associated with the development of EP. Similarly, a retrospective study, Turgut et al., evaluated hematological parameters and found that WBC levels were significantly higher in the EP group than in HP [5]. These findings support the notion that EP involves an inflammatory process.

PLT plays a role in the pathogenesis of EP by participating in processes such as angiogenesis, endothelial damage, and hypoxia [5]. This suggests that PLT levels could serve as a potential early diagnostic indicator for EP. Artunç Ülkümen et al. compared 153 patients with tubal EP and 67 healthy first‐trimester pregnancies. While they did not find a significant difference between the two groups, the mean PLT level was higher in the EP group than in the HP group [22]. Similarly, in a prospective study by Zulfiqar et al., hematological data from 84 patients with cesarean scar pregnancy (CSP) and 88 patients with healthy first‐trimester pregnancies were compared. While no significant difference was found in PLT levels, the mean PLT level was higher in the CSP group than in the HP group [23]. In this study, it was also found that PLT levels were higher in the EP group (*p* = 0.001).

Recent studies also suggest that CBC‐derived markers may help predict methotrexate response in EP. A large retrospective study reported that lower NLR and SII values were associated with higher methotrexate success rates, highlighting a potential role for these markers in treatment planning. While our study focused on diagnosis, this evidence supports the broader clinical relevance of inflammatory indices in EP management [24].

This study also revealed that lymphocyte levels were higher in patients with EP (*p* < 0.001). This finding is noteworthy given the conflicting results in the literature. For instance, Doğru et al. found no significant difference in lymphocyte levels between patients with cesarean scar pregnancy and HP women [25]. In contrast, Eskicioğlu et al. [26] reported higher lymphocyte levels in the EP group but did not find the difference to be statistically significant. The findings obtained in this study, however, suggest a clear association between EP and elevated lymphocyte levels. The cut‐off value for lymphocytes determined by ROC analysis was 1.82 × 10^3^/µL, with a sensitivity of 67.5% and specificity of 43.1%. In the literature, lymphocytes are often evaluated in ratios such as NLR and PLR. However, this study suggests that lymphocyte levels alone could be useful for diagnosing and monitoring EP, warranting further research in this area.

When comparing basophil levels, it was determined that the mean basophil count was higher in the EP group (*p* = 0.005). Basophils are the least abundant subset of leukocytes, constituting less than 1% of total white blood cells, and are primarily involved in allergic responses. However, they also play crucial roles in inflammatory and immune responses. Basophils contain granules filled with vasoactive amines, lipid metabolites, and various cytokines that promote inflammation and the Th2 immune response. A study on mice with systemic lupus erythematosus (SLE), an autoimmune disease, highlighted a notable increase in basophil counts, reinforcing the hypothesis that basophils contribute to the pathogenesis of autoimmune disorders [27]. Given the growing evidence linking EP to autoimmune mechanisms, the results achieved in this study regarding increased basophil levels in EP suggest that autoimmune processes may play a role in its pathophysiology [28]. ROC analysis in this study suggests that basophil levels could serve as a potential biomarker for diagnosing EP. However, larger‐scale studies are required to confirm the generalizability of these findings. The ROC analysis showed that none of the hematologic parameters achieved an AUC above 0.7, indicating limited diagnostic utility. Thus, these markers should be interpreted cautiously and only as adjunctive tools, rather than standalone indicators of EP.

## 5 Limitations

In this study, the higher mean age in the EP group compared to HP is considered a limitation, as age‐related changes may be observed in DNI, PIV, and other inflammatory parameters. In particular, the significant age difference between groups may act as a confounding variable, potentially influencing hematologic markers such as WBC, PLT, and cytokines. Although retrospective design limits statistical adjustment, this imbalance should be taken into account when interpreting the diagnostic value of the parameters. Future prospective studies with age‐matched cohorts are warranted. Additionally, at our hospital, β‐HCG levels are not routinely requested for patients in whom fetal cardiac activity is detected at the time of initial presentation. This posed a challenge in accessing β‐hCG values for the HP group, and consequently, the β‐hCG values of this group could not be included in the study.

## 6 Conclusion

Despite advancements in modern diagnostic and treatment methods, EP remains a significant cause of maternal mortality. When examining the risk factors for EP, advanced maternal age and a history of EP were identified as key contributors. This finding underscores the importance of maintaining a high index of suspicion in patients with these risk factors, as early suspicion is crucial for timely diagnosis. Although high‐resolution ultrasound and serum β‐hCG tests are used for early and accurate diagnosis, challenges in diagnosing certain EP cases persist. While an increase in inflammatory cytokines was observed in EP, hematologic tests may serve as cost‐effective and accessible *supportive tools*, aiding in early clinical suspicion of EP when used in conjunction with imaging and β‐hCG testing, but should not be interpreted as definitive diagnostic measures.

This article was presented as an oral presentation at the 2025 UJOD‐PETKOZ conference in Antalya‐TURKEY.

## Author Contributions

Conceptualization: Dilay Gök Korucu and Şeyma Nur Karadag. Methodology: Dilay Gök Korucu. Validation: Dilay Gök Korucu and Şeyma Nur Karadag. Investigation: Dilay Gök Korucu and Şeyma Nur Karadag. Resources: Dilay Gök Korucu and Şeyma Nur Karadag. Data curation: Dilay Gök Korucu and Şeyma Nur Karadag. Writing – original draft preparation: Dilay Gök Korucu and Şeyma Nur Karadag. Writing – review and editing: Dilay Gök Korucu and Şeyma Nur Karadag. All the authors agreed on the final version of the article. Manuscript has been read and approved by all the authors.

## Funding

The authors received no specific funding for this work.

## Ethics Statement

Karatay University's Medical Faculty Non‐Interventional Clinical Research Ethics Committee on 6 June 2024 (Decision No: 2024/045).

## Conflicts of Interest

The authors declare no conflicts of interest.

## Data Availability

The data that support the findings of this study are available from the corresponding author upon reasonable request.
